# Attenuation Correction Approaches for Serotonin Transporter Quantification With PET/MRI

**DOI:** 10.3389/fphys.2019.01422

**Published:** 2019-11-22

**Authors:** Lucas Rischka, Gregor Gryglewski, Neydher Berroterán-Infante, Ivo Rausch, Gregory Miles James, Manfred Klöbl, Helen Sigurdardottir, Markus Hartenbach, Andreas Hahn, Wolfgang Wadsak, Markus Mitterhauser, Thomas Beyer, Siegfried Kasper, Daniela Prayer, Marcus Hacker, Rupert Lanzenberger

**Affiliations:** ^1^Department of Psychiatry and Psychotherapy, Medical University of Vienna, Vienna, Austria; ^2^Division of Nuclear Medicine, Department of Biomedical Imaging and Image-Guided Therapy, Medical University of Vienna, Vienna, Austria; ^3^QIMP Group, Center for Medical Physics and Biomedical Engineering, Medical University of Vienna, Vienna, Austria; ^4^CBmed, Graz, Austria; ^5^Ludwig Boltzmann Institute Applied Diagnostics, Vienna, Austria; ^6^Division of Neuroradiology and Musculoskeletal Radiology, Department of Biomedical Imaging and Image-Guided Therapy, Medical University of Vienna, Vienna, Austria

**Keywords:** attenuation correction, PET/MRI, serotonin transporter, [^11^C]DASB, occupancy, absolute quantification

## Abstract

**Background:**

Several MR-based attenuation correction (AC) approaches were developed to conquer the challenging AC in hybrid PET/MR imaging. These AC methods are commonly evaluated on standardized uptake values or tissue concentration. However, in neurotransmitter system studies absolute quantification is more favorable due to its accuracy. Therefore, our aim was to investigate the accuracy of segmentation- and atlas-based MR AC approaches on serotonin transporter (SERT) distribution volumes and occupancy after a drug challenge.

**Methods:**

18 healthy subjects (7 male) underwent two [^11^C]DASB PET/MRI measurements in a double-blinded, placebo controlled, cross-over design. After 70 min the selective serotonin reuptake inhibitor (SSRI) citalopram or a placebo was infused. The parameters total and specific volume of distribution (V_T_, V_S_ = BP_P_) and occupancy were quantified. All subjects underwent a low-dose CT scan as reference AC method. Besides the standard AC approaches DIXON and UTE, a T1-weighted structural image was recorded to estimate a pseudo-CT based on an MR/CT database (pseudoCT). Another evaluated AC approach superimposed a bone model on AC DIXON. Lastly, an approach optimizing the segmentation of UTE images was analyzed (RESOLUTE). PET emission data were reconstructed with all 6 AC methods. The accuracy of the AC approaches was evaluated on a region of interest-basis for the parameters V_T_, BP_P_, and occupancy with respect to the results of AC CT.

**Results:**

Variations for V_T_ and BP_P_ were found with all AC methods with bias ranging from −15 to 17%. The smallest relative errors for all regions were found with AC pseudoCT (<|5%|). Although the bias between BP_P_ SSRI and BP_P_ placebo varied markedly with AC DIXON (<|12%|) and AC UTE (<|9%|), a high correlation to AC CT was obtained (*r*^2^∼1). The relative difference of the occupancy for all tested AC methods was small for SERT high binding regions (<|4%|).

**Conclusion:**

The high correlation might offer a rescaling from the biased parameters V_T_ and BP_P_ to the true values. Overall, the pseudoCT approach yielded smallest errors and the best agreement with AC CT. For SERT occupancy, all AC methods showed little bias in high binding regions, indicating that errors may cancel out in longitudinal assessments.

## Introduction

The introduction of combined imaging systems, such as positron emission tomography/computed tomography (PET/CT) proposed a number of benefits, especially for clinical routine, due to aligned structural and molecular information (Townsend et al., 2004). Furthermore, the development of combined positron emission tomography/magnetic resonance imaging (PET/MRI) systems enabled the simultaneous acquisition of functional and molecular information. This option decreases the intrasubject variability between separate measurements (e.g., caused by habituation effects, differences in motivation and attention or fluctuating intrinsic activity) (Hahn et al., 2017). This is especially of importance when functional changes should be compared to molecular changes within the same setting, e.g., after drug challenge (Sander et al., 2013).

However, PET/MRI brought along a major challenge; photons are attenuated to varying extent by different tissue types traversed on the way to the detectors. Ignoring this photon attenuation causes an erroneous reconstruction of the activity distribution (Huang et al., 1979). Hence, it is crucial that PET data is corrected for attenuation. On stand-alone PET systems, attenuation correction (AC) is performed for example with retractable radioactive rod sources of ^68^Ga/^68^Ge, rotating around the patient and generating an AC map (Bailey, 1998). In PET/CT systems, a CT is acquired and scaled from Hounsfield units (HU) to linear attenuation coefficients at 511 keV (Carney et al., 2006). The difficulty with AC in PET/MRI systems is that neither rod sources nor a CT are currently installed due to technical challenges, such as the magnetic field of the MRI (Catana et al., 2013). Another issue is that bone is insufficiently depicted with MRI compared to CT. As gold-standard a separate CT scan would be acquired, further processed and applied for AC on PET/MRI data (Andersen et al., 2014). However, this procedure exposes subjects to additional ionizing radiation and is not practicable for clinical routine. Therefore, several MR-based AC methods have been proposed of which the following are currently implemented as commercial solutions in clinical systems: Siemens Healthineers AG provides solutions such as segmentation of fat and water images (DIXON) (Martinez-Möller et al., 2009) or ultra-short echo time images (UTE) (Catana et al., 2010). General Electric provides a model based approach where a bone model is superimposed on the AC map, derived from segmentation of fat and water images (Sekine et al., 2016). In addition to these commercial approaches, several MR-AC methods have been proposed by the scientific community, such as deep learning algorithms and zero-echo-time sequences (Delso et al., 2015; Gong et al., 2018) or emission-based attenuation correction (Berker and Li, 2016). However, new MR-based AC approaches are commonly based on segmentation or a template/atlas (Ladefoged et al., 2017).

To evaluate the performance of different AC methods, the activity in tissue or the semi-quantitative measure standardized uptake value (SUV) are often used (Schulz et al., 2011; Berker et al., 2012; Burgos et al., 2014; Izquierdo-Garcia et al., 2014; Rausch et al., 2016; Ladefoged et al., 2017). However, in studies with radioligands targeting neurotransmitter proteins, quantification of distribution volumes and binding potentials (BP) are of major interest. This is usually achieved with kinetic modeling and arterial blood and/or a reference region. Ideally, only non-specifically bound radiotracer is found in a reference region (Nørgaard et al., 2019). In this study the radioligand [^11^C]DASB was administered to quantify the serotonin transporter (SERT) protein density in the brain. Kinetic modeling can be performed for example with the simplified reference tissue model (SRTM), a 1 or constrained 2 tissue compartment model, or a graphical analysis (Logan plot) (Ginovart et al., 2001; Nørgaard et al., 2019). An adapted radioligand administration protocol was used in this study, enabling the estimation of the BP with a ratio method (Gryglewski et al., 2017).

Furthermore, drug occupancy is frequently studied for example to elucidate the mechanisms of action of a drug or to determine the appropriate dose, potentially translating to clinical applications (Takano et al., 2016). Thus, longitudinal assessments at baseline and after application of a drug are necessary.

Our main goal was to evaluate the performance of segmentation and atlas-based AC methods in the neurotransmitter system by absolutely quantifying distribution volumes. We hypothesize that differences between the AC approaches of total volume of distribution will be comparable with the results of the literature where SUV and tissue concentration is used, for mathematical reasons (see section “Materials and Methods”). Additionally, we aimed to demonstrate the impact of a bias in the reference region on the specific volume of distribution. Finally, we evaluated the difference between the AC methods when measurements of a longitudinal study are compared.

## Materials and Methods

### Subjects

A total of 18 healthy subjects from a larger ongoing trial were included in this study (7 male, mean age 28.0 ± 9.6 years). To assure the healthy status, all subjects underwent medical examination including blood tests, electrocardiography, neurological and physiological tests as well as a urine drug test at the screening visit. Additionally, all subjects were screened for psychiatric disorders by an experienced psychiatrist with the Structural Clinical Interview according to DSM-IV criteria. A urine pregnancy test was acquired for all female subjects prior to the scans.

### Positron Emission Tomography

All subjects underwent two measurements (mean interval: 36.0 ± 29.6 days) on a PET/MRI system (Siemens Biograph mMR) in a double-blind, placebo-controlled, cross-over design. The radioligand [^11^C]DASB, which specifically binds to the SERT (Wilson et al., 2013), was synthesized as described previously (Haeusler et al., 2009). Radioligand application started outside the scanner, using a syringe pump. The radioligand was infused as bolus for 1 min followed by constant infusion via a cubital vein for a total duration of 180 min. The target dose was calculated as 20 MBq/kg (effective dose for a 75 kg person: 5.55 mSv). PET data was recorded for 120–125 min in list-mode starting 30–45 min post injection. At 70 min post injection the placebo (saline) or 8 mg of the selective serotonin reuptake inhibitor (SSRI) citalopram was infused over 8 min.

### Magnetic Resonance Imaging

Simultaneously with the PET acquisition the standard MR-AC sequences DIXON volume-interpolated breath-hold examination (VIBE, TE_1_/TE_2_/TR = 1.23/2.46/3.60 ms, flip angle = 10°, voxel size 2.6 × 2.6 × 3.1 mm^3^) and ultra-short echo time (UTE, TE_1_/TE_2_/TR = 0.07/2.46/11.94 ms, flip angle = 10°, voxel size 1.6 × 1.6 × 1.6 mm^3^) were recorded. A magnetization prepared rapid gradient echo sequence (MP-RAGE) was acquired for a T1-weighted anatomical image (TE/TR = 4.21/3000 ms, voxel size 1 × 1 × 1.1 mm^3^) for an atlas-based AC approach.

### Attenuation Correction Approaches

Positron emission tomography data was reconstructed with the following AC methods in order to assess their influence on SERT binding based on their availability.

#### CT

According to the EANM guidelines version 2 (Varrone et al., 2009) a regular- or low-dose CT can be used for attenuation correction. All subjects underwent a low-dose CT scan using a PET/CT (Siemens Biograph TruePoint PET/CT, tube potential: 120 kVp, voxel size: 0.59 × 0.59 × 1.5 mm^3^, effective dose: 1 mSv) to keep the radiation exposure as low as possible. For the applicability of the CT scan as AC method, it was preprocessed as described previously (Carney et al., 2006; Ladefoged et al., 2015). In short, the CT scan was co-registered to the structural MRI. Thereafter, it was segmented into bone and non-bone tissues and a bilinear scaling was applied to estimate the linear attenuation coefficients at 511 keV (Kinahan et al., 1998). AC CT was used as reference AC method.

#### DIXON

This approach is based on segmentation of fat and water images originating from in- and opposed-phase images (Dixon, 1984). The final AC map contains the classes soft tissue, fatty tissue, air and if applicable lung tissue (Martinez-Möller et al., 2009).

#### UTE

Two MR images with different ultra-short echo times are segmented into 3 distinctive tissue classes, namely bone, soft tissue and air (Catana et al., 2010).

#### PseudoCT

The pseudoCT was created from an individual structural T1-weighted MRI using an online tool^1^. The method itself is based on a database where pairs of T1-weighted MRIs and co-registered CT scans are stored. The software analyzes the best fitting MRIs, assigns weights and averages the corresponding CTs accordingly. The resulting pseudoCT was then processed equally to the CT (Burgos et al., 2014).

#### RESOLUTE

This AC method is based on AC UTE but additionally segments CSF as well as other tissue classes and uses continuous linear attenuation coefficients for a more accurate AC map (Ladefoged et al., 2015, 2017). For creation of the AC maps an online tool provided by the authors of Ladefoged et al. (2015) was used.

#### Bone Demonstrator

The bone demonstrator (BD) is a non-commercial prototype provided by Siemens Healthineers AG. An MRI, aligned with a bone model, is non-rigidly registered to the individual DIXON MR images. The registered bone model is then superimposed on the AC DIXON with continuous linear attenuation coefficients (Paulus et al., 2015; Koesters et al., 2016).

**FIGURE 1:**
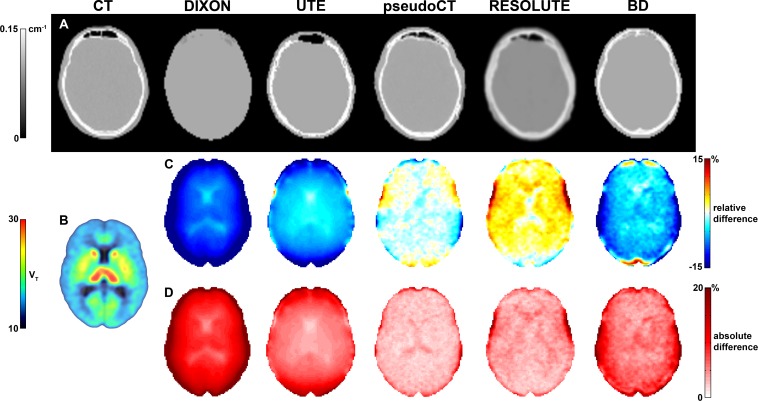
Panel **(A)** depicts the final AC maps of all described AC approaches from one representative subject, panel **(B)** shows the group average V_T_ placebo reconstructed with AC CT, panels **(C,D)** illustrate the voxel-wise group average relative and absolute percentage difference to AC CT-based V_T_. Smaller differences in the center of the brain compared to areas in the proximity to bone are visible.

Figure 1A depicts the final AC maps of one representative subject.

### PET Data Reconstruction and Processing

Three consecutive frames (each 10 min) in tracer equilibrium, starting from 95 min post PET acquisition start, were reconstructed with an ordinary Poisson-ordered subset expectation maximization algorithm (OP-OSEM, 3 iterations, 21 subsets, voxel size 2.09 × 2.09 × 2.03 mm^3^). The data was corrected for dead time, randoms, scatter and attenuation, either with CT, DIXON, UTE, pseudoCT, RESOLUTE or BD. Furthermore, the data was decay-corrected to the start of the radioligand administration to be in accordance with the activity of the blood samples. The offline reconstruction tool was provided by Siemens Healthineers AG.

Subsequently, data was processed with Statistical Parametric Mapping, version 12 (SPM12)^2^ using default algorithms and settings unless indicated otherwise. For motion correction, data was realigned to a mean image (quality = 1) which was co-registered to the T1-weighted structural MR image. Thereafter, the MR image was normalized via tissue probability maps to MNI space. The resulting transformation matrix was then applied to the dynamic PET data. Time-activity values for the 3 mentioned frames were derived from 7 regions-of-interest (ROIs) of the Automated Anatomic Labeling atlas (Tzourio-Mazoyer et al., 2002), namely the temporal lobe, anterior cingulate cortex (ACC), amygdala, caudate, putamen, thalamus and cerebellar gray matter excluding the vermis. The left and corresponding right ROI of each hemisphere were averaged for simplification. The regions were chosen to cover areas across the whole brain with varying amount of SERT binding.

### SERT Quantification

The bolus plus constant infusion protocol enabled the use of an equilibrium method to quantify the total volume of distribution (V_T_), the binding potential in plasma (BP_P_ = V_S_) and the occupancy of the SSRI on the SERT with the 3 consecutive frames. This method has been shown to provide valid estimates of SERT binding without the need for kinetic modeling (Ginovart et al., 2001; Gryglewski et al., 2017). V_T_ is derived from C_T_/C_P_ where C_T_ is the radioligand concentration in tissue, averaged across the 3 frames and C_P_ the radioligand concentration in plasma. C_P_ was calculated as the mean product of whole blood activity, the plasma and the parent fraction in arterial blood samples, drawn 120, 130 and 140 min after start of radioligand infusion. The blood time points were approximately corresponding to the start time points of the PET frames. Radioactivity was measured in whole blood and, after centrifugation, in plasma in a γ-counter (Wizard^2^, 3″, Perkin Elmer). Additionally, the fraction of unchanged [^11^C]DASB was determined using cartridges (Ginovart et al., 2001).

BP_P_ is calculated as V_T_ − V_ND_ where V_ND_ describes the V_T_ of a reference region, i.e., the cerebellar gray matter for [^11^C]DASB (Parsey et al., 2006). Finally, the occupancy was determined as the relative change in BP_P_ between the placebo and the SSRI scan (Innis et al., 2007). All estimated parameters are based on ROIs.

$$\text{Occupancy}\left(\%\right)=\left(1-\left(\frac{{\text{BP}}_{P\,\left(\mathrm{SSRI}\right)}}{{\text{BP}}_{P\,\left(\mathrm{Placebo}\right)}}\right)\right)\times 100\left(1\right)$$

### Statistical Evaluation

To assess the difference between AC methods, the relative difference (RD) of V_T_, BP_P_, and occupancy was calculated with regard to the reference AC approach CT using ROIs.

$$\text{RD}\left(\%\right)=\left(\frac{{\text{Parameter}}_{\text{ACX}}-{\mathrm{Parameter}}_{\text{ACCT}}}{{\text{Parameter}}_{\text{ACCT}}}\right)\times 100\left(2\right)$$

Furthermore, the absolute percentage difference (AD) was calculated as

$$\text{AD}\left(\%\right)=\left(\frac{\left|{\text{Parameter}}_{\text{ACX}}-{\mathrm{Parameter}}_{\text{ACCT}}\right|}{{\text{Parameter}}_{\text{ACCT}}}\right)\times 100\left(3\right)$$

ACX was defined as one of the investigated AC approaches (DIXON, UTE, pseudoCT, RESOLUTE, or BD). Finally, linear regression of V_T_, BP_P_, and occupancy was conducted to detect bias (slope, intercept) and to evaluate similarity (*r*^2^) of the different AC approaches with respect to AC CT.

## Results

**FIGURE 2:**
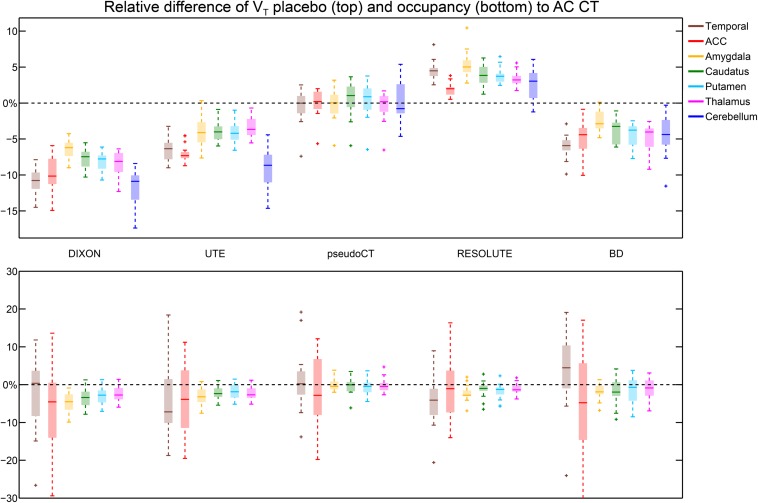
Top, Boxplots depicting the relative difference for each ROI of V_T_ placebo estimated with the proposed AC methods with respect to AC CT. Outliers present for most regions of AC pseudoCT were all from the same subject. Bottom, Boxplots represent the relative difference of each ROI of occupancy compared to AC CT. The figure was limited to –30% for visual purposes. Therefore, the lower whisker of AC BD is just partly visible (actual length: –15 to –34%). Furthermore, three outliers are missing: ACC in AC DIXON (–58%), temporal lobe (–56%), and ACC in AC BD (–91%).

The following paragraphs outline key results for all tested AC methods. A complete list of the group mean relative and absolute percentage differences and standard deviations for V_T_ (placebo, SSRI), BP_P_ (placebo, SSRI) and occupancy with all proposed AC methods compared to the reference AC CT can be found in Tables 1, 2. RD and AD were mostly in a similar range indicating that the bias of a ROI of a specific AC is almost exclusively in one direction across the group. This effect is especially noticeable for the parameter V_T_ (see Table 1). The results of the ROI-based regression between the proposed AC methods and AC CT can be found in Table 3. The values of the SSRI and placebo measurement were merged to have one *r*^2^, slope and intercept per AC approach and parameter. When analyzed separately the *r*^2^ values were rather comparable with V_T_ placebo *r*^2^ ranging from 0.996 to 0.998 in comparison to V_T_ SSRI *r*^2^ ranging from 0.992 to 0996. Similar results were achieved for BP_P_ placebo (*r*^2^ ranging from 0.996 to 0.999) and BP_P_ SSRI (*r*^2^ ranging from 0.983 to 0.992). Furthermore, slope and intercept were similar. Figure 2 depicts boxplots with relative difference of V_T_ placebo and occupancy to the reference AC method CT. For illustration purposes Figure 1B shows the voxel-wise AC CT-based V_T_ placebo averaged across all subjects. Figures 1C,D depict the average voxel-wise relative and absolute percentage differences of V_T_ placebo to AC CT.

**TABLE 1:** Group mean relative difference and standard deviation of V_T_, BP_P_, and occupancy estimated with the proposed AC methods with respect to AC CT for every ROI.

**Mean RD (%)**	**Temporal**	**ACC**	**Amygdala**	**Caudate**	**Putamen**	**Thalamus**	**Cerebellum**
**AC DIXON**							
V_T_ placebo	−11.0 ± 1.7	−9.7 ± 2.3	−6.4 ± 1.4	−7.8 ± 1.4	−8.2 ± 1.4	−8.4 ± 1.6	−11.7 ± 2.6
V_T_ SSRI	−11.1 ± 1.6	−9.2 ± 1.6	−6.1 ± 1.7	−7.0 ± 1.6	−7.6 ± 1.5	−7.7 ± 1.5	−11.8 ± 2.7
BP_P_ placebo	−8.8 ± 4.4	−4.1 ± 7.9	−1.5 ± 3.0	−5.0 ± 1.9	−5.9 ± 1.8	−6.6 ± 1.9	−
BP_P_ SSRI	−6.9 ± 11.2	7.2 ± 21.2	8.8 ± 9.9	1.7 ± 4.8	−1.2 ± 3.4	−1.5 ± 4.2	−
Occupancy	−2.1 ± 9.3	−8.7 ± 16.0	−4.7 ± 2.7	−3.5 ± 2.5	−2.8 ± 2.3	−2.4 ± 2.0	−
**AC UTE**							
V_T_ placebo	−6.5 ± 1.7	−7.1 ± 1.2	−4.1 ± 1.9	−3.9 ± 1.4	−4.2 ± 1.4	−3.5 ± 1.4	−9.0 ± 2.8
V_T_ SSRI	−6.5 ± 2.0	−7.1 ± 1.0	−4.3 ± 2.2	−4.2 ± 1.5	−4.4 ± 1.4	−3.5 ± 1.6	−9.0 ± 2.9
BP_P_ placebo	0.8 ± 5.9	−1.1 ± 7.2	0.3 ± 2.1	−0.3 ± 1.4	−1.0 ± 1.4	−0.4 ± 1.3	−
BP_P_ SSRI	8.7 ± 12.6	7.5 ± 21.7	8.6 ± 9.5	4.7 ± 4.4	3.0 ± 4.0	5.2 ± 4.0	−
Occupancy	−4.4 ± 8.8	−4.3 ± 9.4	−3.0 ± 2.4	−2.3 ± 1.8	−2.0 ± 1.9	−2.4 ± 1.6	−
**AC pseudoCT**							
V_T_ placebo	−0.4 ± 2.2	0.0 ± 1.8	−0.1 ± 2.0	0.4 ± 2.3	0.5 ± 2.4	−0.4 ± 1.9	0.0 ± 3.0
V_T_ SSRI	−0.6 ± 2.2	0.3 ± 1.5	−0.3 ± 2.4	0.4 ± 1.8	0.5 ± 1.8	−0.1 ± 1.7	−0.3 ± 3.0
BP_P_ placebo	−2.0 ± 7.2	0.5 ± 8.2	−0.3 ± 2.8	0.8 ± 3.5	0.8 ± 3.2	−0.6 ± 2.4	−
BP_P_ SSRI	−3.7 ± 13.0	4.4 ± 22.3	−0.2 ± 5.8	1.4 ± 4.2	1.7 ± 3.9	0.4 ± 3.9	−
Occupancy	1.0 ± 7.7	−2.2 ± 8.9	0.0 ± 1.6	−0.3 ± 2.2	−0.4 ± 2.0	−0.3 ± 1.7	−
**AC RESOLUTE**							
V_T_ placebo	4.5 ± 1.3	1.9 ± 0.9	5.3 ± 1.8	3.8 ± 1.3	3.8 ± 1.1	3.4 ± 1.0	2.5 ± 2.2
V_T_ SSRI	4.4 ± 1.5	2.2 ± 0.7	5.2 ± 1.5	4.1 ± 1.1	4.5 ± 1.1	4.2 ± 1.1	2.3 ± 2.1
BP_P_ placebo	10.4 ± 6.0	0.8 ± 7.0	8.0 ± 3.1	4.8 ± 2.3	4.7 ± 1.5	4.0 ± 1.4	−
BP_P_ SSRI	16.6 ± 10.4	4.0 ± 20.3	13.4 ± 6.6	7.5 ± 3.2	8.0 ± 3.3	7.3 ± 3.3	−
Occupancy	−4.2 ± 6.4	−1.2 ± 8.1	−2.2 ± 2.0	−1.3 ± 2.1	−1.7 ± 1.9	−1.4 ± 1.5	−
**AC BD**							
V_T_ placebo	−6.0 ± 1.6	−4.6 ± 2.4	−2.5 ± 1.4	−3.9 ± 1.6	−4.3 ± 1.6	−4.7 ± 1.8	−4.3 ± 2.8
V_T_ SSRI	−6.5 ± 1.6	−4.1 ± 1.7	−2.9 ± 1.7	−3.6 ± 1.8	−4.0 ± 1.7	−4.4 ± 1.7	−5.2 ± 3.2
BP_P_ placebo	−11.1 ± 5.0	−5.7 ± 8.8	−0.9 ± 3.2	−3.6 ± 2.2	−4.4 ± 2.0	−4.9 ± 2.1	−
BP_P_ SSRI	−15.4 ± 10.5	1.2 ± 18.7	2.9 ± 6.5	−1.0 ± 5.9	−2.4 ± 4.4	−3.2 ± 4.9	−
Occupancy	0.9 ± 16.9	−8.3 ± 24.9	−1.9 ± 2.0	−1.5 ± 3.7	−1.3 ± 3.3	−0.8 ± 2.7	−

**TABLE 2:** Group mean absolute percentage difference and standard deviation of V_T_, BP_P_, and occupancy estimated with the proposed AC methods with respect to AC CT for every ROI.

**Mean AD (%)**	**Temporal**	**ACC**	**Amygdala**	**Caudate**	**Putamen**	**Thalamus**	**Cerebellum**
**AC DIXON**							
V_T_ placebo	11.0 ± 1.7	9.7 ± 2.3	6.4 ± 1.4	7.8 ± 1.4	8.2 ± 1.4	8.4 ± 1.6	11.7 ± 2.6
V_T_ SSRI	11.1 ± 1.6	9.2 ± 1.6	6.1 ± 1.7	7.0 ± 1.6	7.6 ± 1.5	7.7 ± 1.5	11.8 ± 2.7
BP_P_ placebo	9.2 ± 3.5	7.0 ± 5.2	2.8 ± 1.6	5.0 ± 1.9	5.9 ± 1.8	6.6 ± 1.9	−
BP_P_ SSRI	10.8 ± 7.2	14.5 ± 16.8	9.2 ± 9.5	3.7 ± 3.4	2.8 ± 2.2	3.4 ± 2.7	−
Occupancy	6.7 ± 6.5	11.7 ± 13.9	4.7 ± 2.7	3.7 ± 2.1	3.0 ± 2.0	2.7 ± 1.6	−
**AC UTE**							
V_T_ placebo	6.5 ± 1.7	7.1 ± 1.2	4.1 ± 1.8	3.9 ± 1.4	4.2 ± 1.4	3.5 ± 1.4	9.0 ± 2.8
V_T_ SSRI	6.5 ± 2.0	7.1 ± 1.0	4.3 ± 2.2	4.2 ± 1.5	4.4 ± 1.4	3.5 ± 1.6	9.0 ± 2.9
BP_P_ placebo	5.1 ± 2.8	6.0 ± 3.9	1.6 ± 1.4	1.1 ± 0.8	1.4 ± 1.1	1.1 ± 0.8	−
BP_P_ SSRI	11.7 ± 9.7	15.1 ± 17.0	9.4 ± 8.8	5.0 ± 4.1	4.0 ± 3.0	5.2 ± 3.9	−
Occupancy	8.3 ± 5.2	8.5 ± 5.6	3.2 ± 2.1	2.4 ± 1.6	2.2 ± 1.6	2.5 ± 1.4	−
**AC pseudoCT**							
V_T_ placebo	1.5 ± 1.7	1.3 ± 1.2	1.5 ± 1.4	1.8 ± 1.4	1.9 ± 1.5	1.2 ± 1.5	2.5 ± 1.6
V_T_ SSRI	1.5 ± 1.7	1.2 ± 0.9	1.7 ± 1.7	1.4 ± 1.1	1.4 ± 1.2	1.2 ± 1.2	2.5 ± 1.6
BP_P_ placebo	5.1 ± 5.3	6.9 ± 4.1	2.0 ± 1.8	2.9 ± 1.9	2.5 ± 2.0	1.6 ± 1.8	−
BP_P_ SSRI	9.2 ± 9.7	16.5 ± 15.2	4.3 ± 3.7	3.6 ± 2.4	3.6 ± 2.1	3.1 ± 2.3	−
Occupancy	5.1 ± 5.7	7.5 ± 4.9	1.3 ± 0.9	1.6 ± 1.5	1.6 ± 1.3	1.3 ± 1.2	−
**AC RESOLUTE**							
V_T_ placebo	4.5 ± 1.3	1.9 ± 0.9	5.3 ± 1.8	3.8 ± 1.3	3.8 ± 1.1	3.4 ± 1.0	2.8 ± 1.8
V_T_ SSRI	4.4 ± 1.5	2.2 ± 0.7	5.2 ± 1.5	4.1 ± 1.1	4.5 ± 1.1	4.2 ± 1.1	2.6 ± 1.6
BP_P_ placebo	10.5 ± 5.9	5.4 ± 4.5	8.0 ± 3.1	5.0 ± 1.9	4.7 ± 1.5	4.0 ± 1.4	−
BP_P_ SSRI	16.6 ± 10.4	13.7 ± 15.2	13.4 ± 6.6	7.5 ± 3.2	8.0 ± 3.3	7.3 ± 3.3	−
Occupancy	6.0 ± 4.7	6.8 ± 4.3	2.6 ± 1.5	1.7 ± 1.7	2.0 ± 1.6	1.7 ± 1.1	−
**AC BD**							
V_T_ placebo	6.0 ± 1.6	4.6 ± 2.4	2.5 ± 1.4	3.9 ± 1.6	4.3 ± 1.6	4.7 ± 1.8	4.3 ± 2.8
V_T_ SSRI	6.5 ± 1.6	4.1 ± 1.7	2.9 ± 1.7	3.6 ± 1.8	4.0 ± 1.7	4.4 ± 1.7	5.2 ± 3.2
BP_P_ placebo	11.1 ± 5.0	8.3 ± 6.2	2.6 ± 1.9	3.6 ± 2.2	4.4 ± 2.0	4.9 ± 2.1	−
BP_P_ SSRI	16.1 ± 9.3	14.3 ± 11.5	5.0 ± 5.0	4.2 ± 4.0	3.8 ± 3.3	4.7 ± 3.4	−
Occupancy	10.9 ± 12.7	16.2 ± 20.5	2.1 ± 1.7	3.1 ± 2.3	2.7 ± 2.3	2.3 ± 1.6	−

**TABLE 3:** Region-of-interest-based linear regression parameters (*r*^2^, slope, and intercept) for V_T_, BP_P_, and occupancy estimated with the proposed AC methods with respect to AC CT. The values of the placebo and SSRI scan were merged.

**Regression to AC CT**	***r*^2^**	**Slope**	**Intercept**
**AC DIXON**			
V_T_	0.997	0.93	–0.34
BP_P_	0.997	0.93	0.27
Occupancy	0.898	1.00	–2.10
**AC UTE**			
V_T_	0.998	0.98	–0.55
BP_P_	0.998	0.98	0.23
Occupancy	0.929	0.92	2.93
**AC pseudoCT**			
V_T_	0.997	1.01	–0.10
BP_P_	0.997	1.00	0.00
Occupancy	0.945	1.00	–0.38
**AC RESOLUTE**			
V_T_	0.999	1.04	–0.04
BP_P_	0.998	1.04	0.20
Occupancy	0.948	0.98	–0.21
**AC BD**			
V_T_	0.998	0.96	–0.11
BP_P_	0.997	0.96	0.02
Occupancy	0.841	1.09	–6.40

### DIXON

V_T_ showed the highest RD in the temporal lobe and the cerebellum with underestimations of around 11% and *r*^2^ ∼ 1. The errors of the placebo and SSRI scan were similar for V_T_. In contrast, for BP_P_ the difference was markedly non-uniform between the ROIs and varied between placebo and SSRI scans. The most pronounced difference was found in the amygdala (placebo: −2%, SSRI: 9%). The bias for occupancy was less than −5%, except for the ACC with an error of approximately −9%. Regression confirmed the underestimation with a slope below 1 for V_T_ and BP_P_.

### UTE

Similar to V_T_ obtained with AC DIXON, the largest RD was found in the cerebellum (−9%). The error of BP_P_ varied between the placebo and SSRI scan. For the placebo scan the RD was as low as −1% whereas the SSRI scan showed an overestimation of 9% in the amygdala. The occupancy varied between −4 and −2% in all investigated regions.

### PseudoCT

For V_T_, the AD across all regions was less than 3%. The RD for BP_P_ was similar for the placebo and the SSRI scans with the exception of the ACC, where the placebo scan overestimated the BP_P_ by 1% and the SSRI scan by 4%. The relative variation of the occupancy was between 0 and 1%. However, the AD revealed an increased bias in all estimated parameters, especially for BP_P_ in low binding regions. Nevertheless, the results of all parameters were similar to the ones obtained with AC CT (*r*^2^ ranging from 0.95 to 1). Outliers found in Figure 2, top, were all from the same subject. However, no abnormalities were observed in the pseudoCT AC map, thus, the subject was retained in all analyses.

### RESOLUTE

V_T_ and BP_P_ were overestimated in all regions. The largest deviation for V_T_ was 5% in the amygdala whereas the largest RD for BP_P_ was found in the temporal lobe (17%). The overestimations were also observed with regression (slope: 1.04, intercept 0.20). In contrast, the occupancy was underestimated in all regions by 1% (caudate) to 4% (temporal lobe). The slope and intercept of the regression was almost in alignment with the line of identity (slope between 0.98 and 1.04, intercept between −0.21 and 0.20) and had an *r*^2^ ranging from 0.95 to 1 for all parameters.

### BD

The largest relative errors were found in the low binding region of the temporal lobe (V_T_ SSRI −7%, BP_P_ SSRI −15%). In all other areas, the bias varied between −6 and 1%. The RD of the occupancy was between −2 and 1% with an exception of −8% in the ACC. Again, compared to RD, AD was increased in low binding regions for the parameter BP_P_ as well as occupancy. Regression showed the lowest agreement for occupancy with *r*^2^ of 0.84 slope = 1.09) and intercept = −6.40.

Overall, the agreement of V_T_ and BP_P_ calculations when using different AC methods was close, with *r*^2^ of approximately 1 for the placebo and the SSRI scans. In almost all regions, the smallest relative error was found for AC pseudoCT without mean relative bias in the cerebellum and ACC for V_T_ placebo. However, AD revealed marked errors in low binding regions which were hidden in the RD alone. The regression of AC RESOLUTE and AC pseudoCT showed a slope and intercept similar to the line of identity. The standard deviation was highest for occupancy with AC BD and lowest with AC RESOLUTE in the temporal lobe and ACC, ranging from ±6 to ±16% and ±8 to ±24%, respectively.

## Discussion

In this study, we analyzed the performance of different PET/MRI AC methods, namely, DIXON, UTE, pseudoCT, RESOLUTE and BD with respect to the CT-reference for the quantification of SERT binding and occupancy. In contrast to most of the previous reports we did not use tissue activity or SUV values but focused on different outcome parameters obtained from absolute quantification.

First, we evaluated the performance of the tested AC methods with regards to the parameter V_T_ in comparison to the reference AC CT. When using AC DIXON, V_T_ was underestimated with a regional variability. While subcortical regions close to the center of the brain showed a lower bias, a larger RD was observed in areas close to the skull. This phenomenon and similar RDs were reported by other groups (Izquierdo-Garcia et al., 2014; Rausch et al., 2017) and demonstrated on a voxel-wise basis (Ladefoged et al., 2017). The bias is likely caused by the missing bone information (Andersen et al., 2014).

The lack of bone can be accounted for to some degree by superimposing a bone segment on the AC DIXON map (i.e., the BD method). The improvement approximately halved the errors in all regions. However, since AC BD is based on AC DIXON, errors in the latter are also transferred to BD (e.g., filled air cavities) (Rausch et al., 2017). Similarly, AC UTE offered an improvement to the RD of V_T_, although still with higher underestimations in the vicinity of the skull. The error arises from segmentation misclassifications in the tissue/air border areas (Aasheim et al., 2015). Discontinuities of the segmented skull and neglect of regional variation in bone density are further issues leading to inaccurate PET quantification (Ladefoged et al., 2015). However, overall, the bias was smaller than with AC DIXON and in some regions comparable to AC BD, presumably due to the additional bone segment. In AC RESOLUTE the segmentation of UTE images was optimized with additional tissue classes and masks in areas difficult to segment, such as the nasal septa, ethmoidal-, frontal sinuses and mastoid process. Another difference is that continuous linear attenuation coefficients are used for a more accurate attenuation map (Ladefoged et al., 2015). This improved the V_T_ values, however, yielded overestimations in all regions. Our results deviate slightly from those of Ladefoged et al. (2015), who reported underestimations in some areas. However, the same group published a larger study with errors similar to the ones found in this work (Ladefoged et al., 2017). The best performance of V_T_ in terms of bias was found with the atlas-based AC method pseudoCT, yielding high accuracy in all regions. As a limitation for this (Burgos et al., 2016) and similar approaches (Mérida et al., 2017) it is worth to mention the necessity of a representative MRI-CT database. Of note, similar RD and AD are expected between tissue concentration and V_T_ values since V_T_ mathematically equals the tissue concentration divided by the radioligand concentration in plasma.

The more commonly used outcome parameter for SERT and other neurotransmitter applications is BP_P_. By definition, the difference between BP_P_ and V_T_ can only arise from the reference region (here the cerebellar gray matter). It is therefore important to note that the error of the AC methods is markedly non-uniform across the brain, particularly for those methods without or with only limited modeling of bone tissue. Regions in proximity of bone (e.g., cerebellum) show a higher bias than regions in the center of the brain (e.g., striatum). Hence, outcome parameters, which are dependent on interregional differences (e.g., BP_P_) or ratios (distribution volume ratio) will incorporate the non-uniform error as additional source of bias. Another issue is that SSRIs decrease the binding also in the reference region. Although the cerebellar gray matter was identified as the optimal reference region for [^11^C]DASB, a displacement of up to 33% was described after an acute sertralin challenge (Parsey et al., 2006; Turkheimer et al., 2012). However, this will not affect the comparison between the different AC methods as differences in the reference region will be equally present for all AC approaches. Nonetheless, this may indeed have an effect when using outcome parameters such as BP_P_ or BP_ND_, which require an unbiased reference region. Here, lower binding or in other words small numerical uncertainties will translate into larger errors of percentage. This is a potential explanation for the differences between the SSRI and placebo scans observed for BP_P_ when using DIXON and UTE.

Differently to this study, binding potentials are also commonly estimated with kinetic modeling. Inhomogeneous tracer uptake over time might induce uncertainties in ROIs which can influence the shape of time activity curves and therefore the outcome of the kinetic modeling (Mérida et al., 2017). However, Mansur et al. compared the AC approach pseudoCT to AC CT with the reference kinetic model SRTM as well as an arterial blood-based two tissue compartment model (2TCM) (Mansur et al., 2018). They reported a strong correlation of non-displaceable BP and V_T_ and mostly similar differences (<5%) as (Mérida et al., 2017), who used comparable AC approaches. As similar bias to the ratio method with AC pseudoCT was found here, it might be concluded that the other AC methods of this study yield comparable differences as BP_P_ when kinetic modeling is applied.

Given its clinical importance, SERT occupancy induced by i.v. SSRI challenge was also assessed for all AC methods. Of note, the error decreased in almost all areas compared to V_T_ and BP_P_ thus, indicating that the inaccuracy cancels out, except for errors in the ACC with AC DIXON and BD. For high binding regions of [^11^C]DASB, such as putamen, thalamus and caudate, RD did not exceed errors of −4% with reasonable SD. This is probably based on the small test–retest variability between the placebo and SSRI scan in terms of AC reproducibility since RD and AD are almost identical in both measurements. Therefore, our results might be translatable to longitudinal or occupancy studies, were two measurements are brought into context, independent of the tracer of choice.

We could demonstrate that the bias is not only dependent on the AC method but also on the investigated parameter, which is a complementary discovery to (Ladefoged et al., 2017). Interestingly, a high *r*^2^ was obtained for V_T_ which may enable the rescaling of biased parameters to the true values achieved with AC CT on an ROI-basis. This would enable the usage of all presented AC methods, depending on the scientific question, e.g., investigation of V_T_ in subcortical regions with [^11^C]DASB. However, issues might occur with other parameters such as BP_P_ or occupancy with AC DIXON or BD. The lower *r*^2^ of the occupancy caused by fluctuating errors between the SSRI and placebo scans of BP_P_ limit the applicability of both AC methods to ROIs in the brain center and parameters independent from other regions (e.g., V_T_). Furthermore, we would like to mention that the scalability does not apply to voxel level with the same accuracy (Ladefoged et al., 2017). It also has to be mentioned that a cohort of young adults was investigated (mean age 28.0 ± 9.6 years). Different results might be achieved in elderly or diseased subjects due to varying characteristics that cannot be reflected by the presented AC approaches (e.g., differences in skull density or cortical thickness). Another limitation observed in AC DIXON is the inversion of fat and water images, which leads to incorrect segmentation classes (Ladefoged et al., 2014). The tissue inversion occurs either partially or measurement specific and not subject specific, which is especially an issue in longitudinal studies. It is further an issue for AC BD as it is based on AC DIXON. Hence, the error propagates to AC BD. The impact of tissue inversion on BP_P_ and occupancy was not evaluated due to a lack of data. Another limitation was the necessity for large ROIs in low binding regions which are mostly cortical structures for [^11^C]DASB. This was essential to calculate reliable BP_P_ as these regions have almost as little binding as the reference region (i.e., cerebellum). Additionally, since the AC induced bias is non-uniform, large ROIs, spreading from near the center of the brain to the proximity of bone tissue, might average out some regional effects. Hence, dissimilar results might be achieved in cross-sectional studies for the parameters V_T_ and BP_P_ with tracers with a different distribution.

## Conclusion

In this study, we presented the accuracy of different AC approaches for different binding parameters quantified with [^11^C]DASB. We could show the impact of AC on parameters relying on the accurate quantification of non-displaceable binding (such as BP_P_) and a decreased error if two measurements are brought into context (occupancy), for almost all investigated regions. The AC method pseudoCT performed best compared to AC CT since all examined regions and parameters showed acceptable variations within a range of 4%. Based on these results, AC pseudoCT should be considered as a reliable alternative to AC CT.

## Data Availability Statement

The datasets generated for this study are available from the corresponding author upon request.

## Ethics Statement

The study was approved by the Ethics Committee of the Medical University of Vienna and procedures were carried out in accordance with the Declaration of Helsinki. After a detailed explanation of the study all subjects gave written informed consent.

## Author Contributions

LR, GG, MK, AH, WW, MM, and RL designed the study. GG recruited subjects and medically assisted the measurements. NB-I and WW synthesized the radioligand. LR, GJ, and MK acquired the data. HS and MM analyzed the blood samples. LR, IR, and AH performed data analysis. MHB and SK were medical supervisors. RL was the principal investigator and scientific supervisor of the study. All authors contributed in writing and reviewing the manuscript.

## Conflict of Interest

Siemens Healthineers AG provided the Bone Demonstrator software as well as dedicated reconstruction software through a research agreement with the Medical University of Vienna. The authors declare that the research was conducted in the absence of any commercial or financial relationships that could be construed as a potential conflict of interest.

## Footnotes

1 http://niftyweb.cs.ucl.ac.uk/program.php?p=PCT

2 www.fil.ion.ucl.ac.uk/spm/software/spm12/
